# Dissolution of Zinc Oxide Nanoparticles in the Presence of Slow Acid Generators

**DOI:** 10.3390/ma15031166

**Published:** 2022-02-02

**Authors:** Ronny Kürsteiner, Maximilian Ritter, Yong Ding, Guido Panzarasa

**Affiliations:** Wood Materials Science, Institute for Building Materials, ETH Zürich, Laura-Hezner-Weg 7, 8093 Zürich, Switzerland; ronnyk@ethz.ch (R.K.); maxritter@ethz.ch (M.R.); yoding@ethz.ch (Y.D.)

**Keywords:** zinc oxide, propanesultone, gluconolactone, slow acid generation, dissolution, nanoparticles, programmable assembly and disassembly, pH

## Abstract

We describe a preliminary investigation of the dissolution dynamics of zinc oxide nanoparticles in the presence of cyclic esters (δ-gluconolactone and propanesultone) as slow acid generators. The particles dissolution is monitored by means of turbidimetry and correlated with the evolution of pH over time. The results could be of interest for the design of chemically programmable colloidal systems.

## 1. Introduction

Investigating the dissolution of inorganic nanoparticles is a topic of great relevance, both theoretical and practical, with applications ranging from advanced materials to environmental science and medicine [1,2,3,4]. Cyclic esters are slow acid generators, useful for controlling the evolution of pH in time due to the acids produced by their hydrolysis [5]. As such, they are important tools for the application of systems chemistry principles to materials science [6,7]. In the context of our research on the chemical programming of material systems, we became interested in applying the same principles to inorganic materials, in addition to supramolecular and polymeric ones [8,9,10,11,12,13,14,15]. We began by studying the dissolution dynamics of inorganic nanoparticles in the presence of slow acid generators. For our preliminary investigation, we chose zinc oxide ZnO nanoparticles as a model system for its great relevance [16,17,18,19,20,21,22,23] and commercial availability. As slow acid generators, we chose a lactone, δ-gluconolactone (GL), and a sultone, 1,3-propanesultone (PrS). The kinetics of hydrolysis of both are already known, and their reaction with water (Equations (1) and (2)) give, respectively, gluconic acid (pK_a_ 3.86, a typical carboxylic acid) [5] and 1-hydroxypropanesulfonic acid (pK_a_ 1.53, a typical sulfonic acid) [10], which are both strong enough to dissolve zinc oxide, thus forming the corresponding soluble salts (Equations (1) and (2)):


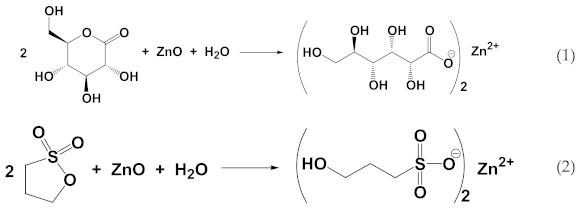


We studied the dissolution of ZnO nanoparticles by measuring the evolution of pH and turbidity over time, keeping constant the concentration of ZnO (5 mM) and varying that of the slow acid generators (50, 100, or 200 mM). With a lower (1 mM) ZnO concentration, the dissolution was too fast (especially with GL) to ensure proper turbidimetry measurements. 

## 2. Materials and Methods

Zinc oxide dispersion (nanoparticles, <100 nm particle size (TEM), ≤40 nm avg. part. Size (APS), 20 wt.% in H_2_O), δ-gluconolactone (GL, meets USP testing specifications), and 1,3-propanesultone (PrS, 98%) were purchased from Sigma-Aldrich, St. Louis, MI, USA. Unless otherwise stated, all chemicals were of an analytical or reagent grade purity and used as received. Water was purified by means of a MilliQ system (resistivity ≥ 18 MΩ). All the experiments were performed at room temperature (23 ± 1 °C).

For pH measurements, a Hanna Instruments (Woonsocket, RI, USA) HI5222-02 benchtop pH-meter was used together with a HI1330B glass body combination pH microelectrode from the same company. The pH-meter was calibrated with standard buffer solutions (pH values: 1.68, 4.01, 7.01, 10.01, and 12.45) before each set of analysis. The pH-electrode was cleaned after each analysis by repeated immersion in water, the excess water gently was removed with hairless paper and immediately immersed in the solution to be analyzed. The pH-meter was interfaced with a computer through the software HI92000–5.0.38 (Hanna Instruments, Woonsocket, RI, USA) to allow continuous recording of pH values with a time interval of 2 s. The pH measurements were carried out on a 10-mL reaction mixture, in 15-mL glass vials with stirring of 500 rpm. 

Turbidimetry measurements were performed with a PerkinElmer (PerkinElmer Life and Analytical Sciences, 710 Bridgeport Avenue Shelton, CT 06484-4794 USA) LAMBDA 650 UV-visible spectrophotometer using a quartz cuvette with an optical path of 1 cm. The content of the cuvette was stirred (500 rpm) with a suitable magnetic bar. The measurements were performed at a fixed wavelength (600 nm) with a 30-s time interval. For the experiments with PrS, 12 µL of the ZnO dispersion were diluted in a vial with 8 mL of water. The PrS was dissolved in water (total volume 2 mL) by sonication, and added to the ZnO dispersion. For the experiments with GL, 12 µL of the ZnO dispersion were diluted in a vial with 9 mL of water. The GL was dissolved in 1 mL of water by sonication, and added to the ZnO dispersion. In both cases, the mixture was then transferred to the cuvette under stirring and the measurement started, in any case with a delay ≤ 60 s from the addition of the slow acid generator (PrS or GL). 

Transmission electron microscopy (TEM) was performed with a Jeol JEM 1400 instrument at 120 kV acceleration voltage. To prepare the sample, a drop of dilute particle suspension was deposited on a carbon-coated 400-mesh sized copper grid and air dried.

X-ray powder diffraction (XRPD) was performed with a Panalytical X’Pert PRO MPD using Cu Kα1 radiation in Bragg–Brentano geometry. The ZnO suspension was centrifuged and air dried, then the powdered sample was placed on a zero-background sample holder and measured from 5° to 80° 2θ with a step size of 0.0668° and a scan speed of 0.034° s^−1^ whilst continuously spinning at 15 min^−1^ and maintaining an X-ray footprint of 10 × 10 mm^2^. The crystallite size was estimated using Scherrer’s equation [24], assuming a Scherrer formfactor of 1.

Dynamic light scattering (DLS) measurements were performed with a Malvern Zetasizer Nano ZS, employing a non-invasive back scatter detection system at a 173° detection angle. Prior to measurements, the ZnO dispersions were diluted by a factor of 20 to mitigate further dissolution and adjust the count rate. The measurements consisted of 10 runs of 10 s each and were performed in disposable polystyrene cuvettes at 25 °C. The refractive indices of sample and dispersant were set to 1.99 and 1.33, respectively. The viscosity of the dispersant was set to 0.8872 cP.

## 3. Results and Discussion

**Figure 1:**
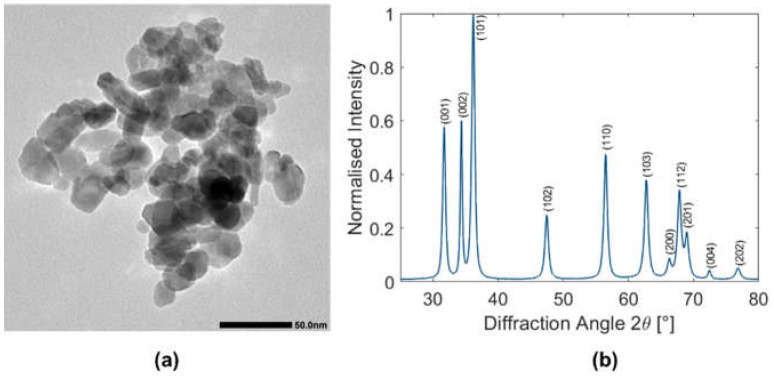
Representative (**a**) TEM image (scale bar 50 nm) and (**b**) XRPD pattern of the ZnO nanoparticles used for the experiments.

The commercial ZnO nanoparticle dispersion was characterized by means of transmission electron microscopy (TEM; Figure 1a and Figure S1) and X-ray powder diffraction (XRPD, Figure 1b). The results are in good agreement with the data given by the producer. TEM shows relatively polydisperse spheroidal and plate-like particles, with an average crystallite size around 13 ± 1 nm (estimated from X-ray diffraction data).

**Figure 2:**
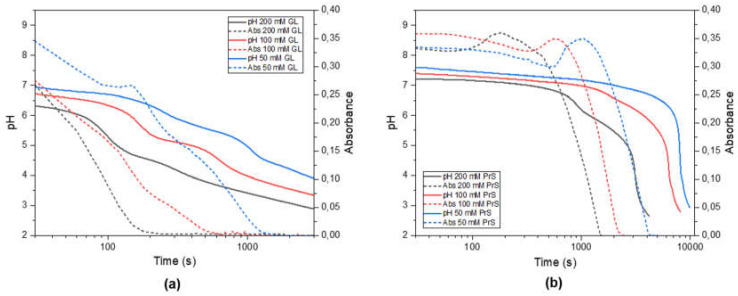
Evolution of pH and turbidity over time for a 5 mM ZnO suspension with different concentrations of slow acid generators: (**a**) δ-gluconolactone and (**b**) 1,3-propanesultone.

The hydrolysis of GL is much faster than that of PrS. Nevertheless, in the range of concentrations (50–200 mM) and over the timescales (<3 h) investigated here, the hydrolysis of both GL and PrS occurs approximately at a constant rate [10]. In the absence of pH-buffering effects, this results in a steady decrease of pH, as shown in Figure S2. On the other hand, in presence of ZnO, the pH-time evolution of both GL and PrS is characterized by two inflection points (Figure 2, solid lines), which are more evident the higher the concentration of cyclic ester. 

From the associated turbidimetry plots (Figure 2, dotted lines) it is possible to relate the observed pH changes with the ZnO dissolution. In the system ZnO-GL (Figure 2a), there is a constant, almost linear decrease in turbidity, with inflection points which follow the trend of those observed in the pH curves. In the system ZnO-PrS, by contrast, initially the decrease in turbidity is very slow and is even followed by a transient increase, after which it decreases very rapidly. In addition, in this case there is a good agreement with the temporal evolution of pH, except for these transient increases in turbidity, whose maxima are all in correspondence of a pH ≈ 7 for all three PrS concentrations. This increase in turbidity could be due to the formation of particle aggregates. Dynamic light scattering (DLS) indeed showed a dramatic increase in the hydrodynamic particle size, however with great delay, which is after the transient increase in turbidity that was observed in the spectrophotometer (Figure S3). This delay could have been due to the different experimental conditions (sample more diluted and unstirred). The mechanism leading to a transient aggregate formation is not yet clear, however it is plausible to hypothesize that the steady acid generation would not only cause particle dissolution but also interfere with their surface charge, thus reducing their electrostatic repulsion. Zeta potential titration has been used to attempt to validate this hypothesis, however the results were not conclusive. Another hypothesis would involve the preferential dissolution of certain crystal planes, facilitating the oriented attachment of the particles. 

Although preliminary, these observations allow one to conclude that the dissolution of zinc oxide nanoparticles can be achieved using slow acid generators, and that the dynamics of the process are strongly influenced not only by their concentrations but especially by their nature. More accurate insight will be obtained by repeating these experiments using particles of a controlled size and size distribution, shape, crystallinity, and chemical composition. Measuring the concentration of zinc ions in solution as a function of time, together with the evolution of pH, could help in further understanding the particle dissolution dynamics. Given the versatility of slow acid generators for the programming of pH-controlled colloidal systems, their application to inorganic nanoparticles could provide interesting opportunities. 

## Data Availability

Not applicable.

## Supplementary Materials

The following are available online at https://www.mdpi.com/article/10.3390/ma15031166/s1, Figure S1: Representative TEM images of the ZnO nanoparticles used for the experiments. Figure S2: pH-Time curves for different concentrations of (a) δ-gluconolactone, (b) propanesultone in pure water. Figure S3: DLS curves for the system ZnO-PrS.
