# Supplementary material for: Dissolution of Zinc Oxide Nanoparticles in the Presence of Slow Acid Generators
Source: Materials (Basel). 2022 Feb 2;15(3):1166. doi: 10.3390/ma15031166 (PMC8838790; doi:10.3390/ma15031166)
Supplement: Supplementary file 1 [file materials-15-01166-s001.zip › materials-1573198-supplementary.pdf]

materials

Supplemental Materials

Dissolution of Zinc Oxide Nanoparticles in the Presence of Slow Acid Generators

Ronny Kürsteiner, Maximilian Ritter, Yong Ding and Guido Panzarasa *

Wood Materials Science, Institute for Building Materials, ETH Zürich, Laura-Hezner-Weg 7, 8093 Zürich, Switzerland; ronnyk@ethz.ch (R.K.); maxritter@ethz.ch (M.R.); yoding@ethz.ch (Y.D.)
* Correspondence: guidop@ethz.ch

Citation: Kürsteiner, R.; Ritter, M.; Ding, Y.; Panzarasa, G. Dissolution of Zinc Oxide Nanoparticles in Presence of Slow Acid Generators. *Materials* **2022**, *15*, 1166. https://doi.org/10.3390/ma15031166

Academic Editor: Alina Pruna

Received:11 January 2022
Accepted:30 January 2022
Published: 2 February 2022

Publisher's Note: MDPI stays neutral with regard to jurisdictional claims in published maps and institutional affiliations.

Copyright: © 2022 by the authors. Licensee MDPI, Basel, Switzerland. This article is an open access article distributed under the terms and conditions of the Creative Commons Attribution (CC BY) license (https://creativecommons.org/licenses/by/4.0/).

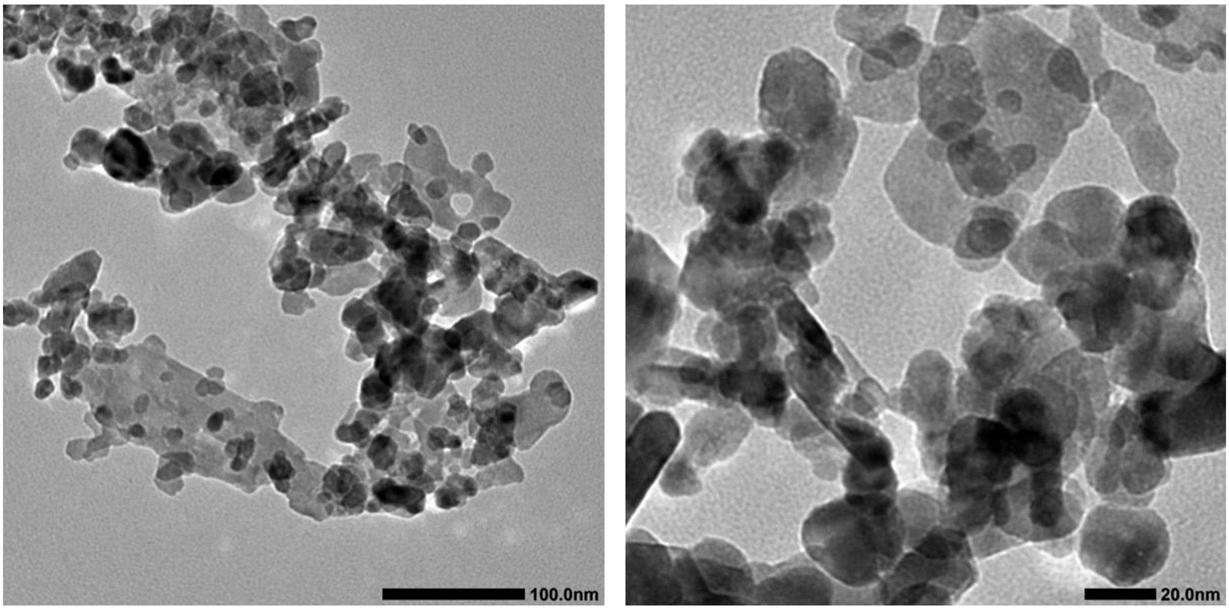

Figure S1. Representative TEM images of the ZnO nanoparticles used for the experiments.

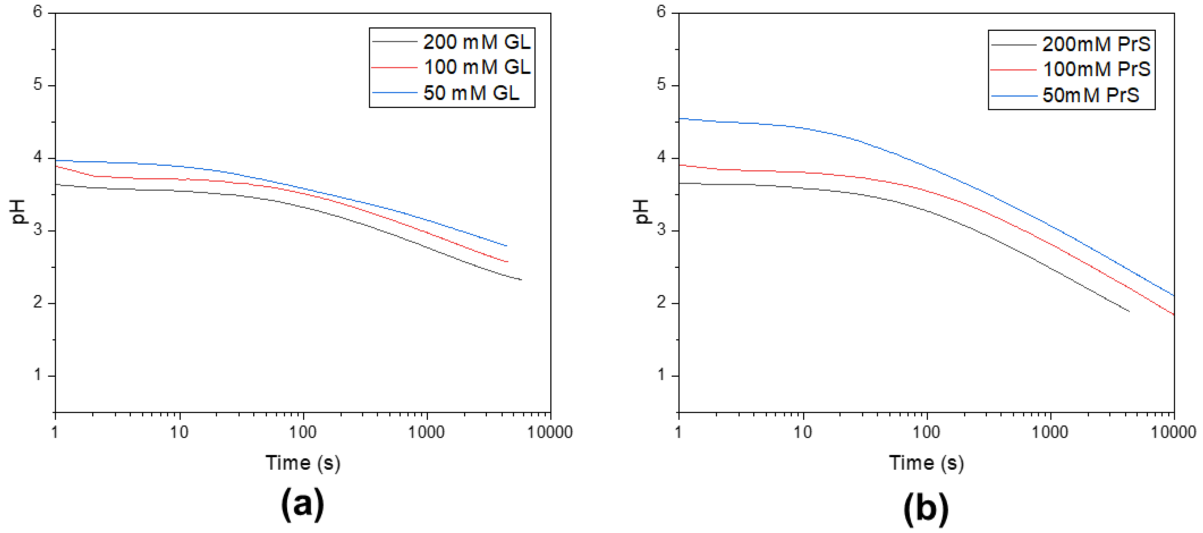

Figure S2. pH-Time curves for different concentrations of (a) δ-gluconolactone, (b) propanesultone in pure water.

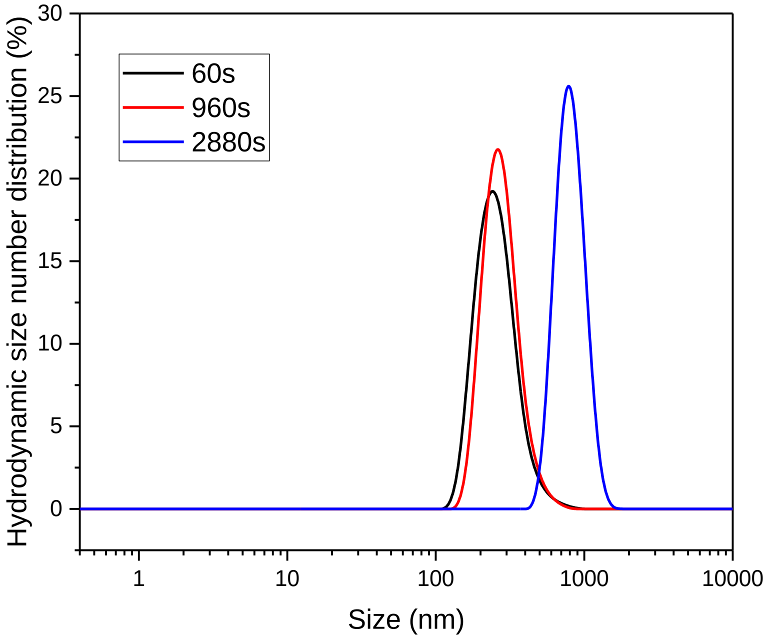

Figure S3. DLS curves for the system ZnO-PrS.
